# IMPORTANCE OF PROMPT CREATINE KINASE MONITORING IN DIABETIC EMERGENCIES

**DOI:** 10.51894/001c.122873

**Published:** 2024-08-30

**Authors:** Apoorv Tiwari, Melanie Corsten, Syeda Salari, Nikhale Malik, Abed Dabaja, Chadi Saad

**Affiliations:** 1 Internal Medicine Garden City Hospital

## 26

### INTRODUCTION

Rhabdomyolysis results from skeletal muscle necrosis, causing the release of intracellular contents into the bloodstream. Research has linked hyperglycemia to it; however, it seldom leads to hemodialysis. Not only does acute kidney injury (AKI) lead to increased mortality rates but also increases overall length of stay in the hospital and healthcare costs. Monitoring creatine kinase at the right moment can contribute to preventing uremic crisis and easing the financial burden.

### CASE DESCRIPTION

We detail a case of 37-year-old male found at his home by the EMS in stuporous state, lying supine on floor, normothermic who was admitted in a hyperosmolar hyperglycemic state accompanied by hypernatremia and azotemia. Upon arrival at our emergency department, he displayed severe dehydration, a blood glucose level surpassing 2,000 mg/dl, and high anion gap metabolic acidosis secondary to acute kidney injury. He was diagnosed to have acute pancreatitis on the basis of CT abdomen and elevated lipase levels. His condition worsened when his creatine kinase levels surged from 210 U/L to 39,000 U/L, leading to oliguric renal failure and the need for hemodialysis. Comprehensive treatment included crystalloids, parenteral insulin, and rigorous ICU monitoring with mechanical ventilation. Following this, the patient showed rapid improvement and was discharged without requiring further dialysis. A thorough history and observation established a rare but strong link between rhabdomyolysis and hyperosmolar hyperglycemic states.

### DISCUSSION/CONCLUSION

Uncontrolled hyperglycemia and hyperosmolarity can intensify nephrotoxic events, especially in patients already facing severe dehydration and prerenal injury. This case emphasizes the critical need for prompt ordering of creatine kinase levels in diabetic emergencies when renal parameters and/or urine output are deteriorating despite adequate fluid resuscitation. Such monitoring ensures early detection and facilitates effective management of rhabdomyolysis-induced AKI, reducing potential complications and improving patient outcomes.

